# Assembly and Capsid Expansion Mechanism of Bacteriophage P22 Revealed by High-Resolution Cryo-EM Structures

**DOI:** 10.3390/v15020355

**Published:** 2023-01-26

**Authors:** Hao Xiao, Junquan Zhou, Fan Yang, Zheng Liu, Jingdong Song, Wenyuan Chen, Hongrong Liu, Lingpeng Cheng

**Affiliations:** 1 Institute of Interdisciplinary Studies, Key Laboratory for Matter Microstructure and Function of Hunan Province, Key Laboratory of Low-dimensional Quantum Structures and Quantum Control, Hunan Normal University, Changsha 410082, China; 2 Kobilka Institute of Innovative Drug Discovery, School of Medicine, Chinese University of Hong Kong, Shenzhen 518172, China; 3 State Key Laboratory of Infectious Disease Prevention and Control, National Institute for Viral Disease Control and Prevention, Chinese Center for Disease Control and Prevention, Beijing 100052, China

**Keywords:** bacteriophage P22, virus assembly, scaffolding protein, capsid expansion, cryo-EM

## Abstract

The formation of many double-stranded DNA viruses, such as herpesviruses and bacteriophages, begins with the scaffolding-protein-mediated assembly of the procapsid. Subsequently, the procapsid undergoes extensive structural rearrangement and expansion to become the mature capsid. Bacteriophage P22 is an established model system used to study virus maturation. Here, we report the cryo-electron microscopy structures of procapsid, empty procapsid, empty mature capsid, and mature capsid of phage P22 at resolutions of 2.6 Å, 3.9 Å, 2.8 Å, and 3.0 Å, respectively. The structure of the procapsid allowed us to build an accurate model of the coat protein gp5 and the C-terminal region of the scaffolding protein gp8. In addition, interactions among the gp5 subunits responsible for procapsid assembly and stabilization were identified. Two C-terminal α-helices of gp8 were observed to interact with the coat protein in the procapsid. The amino acid interactions between gp5 and gp8 in the procapsid were consistent with the results of previous biochemical studies involving mutant proteins. Our structures reveal hydrogen bonds and salt bridges between the gp5 subunits in the procapsid and the conformational changes of the gp5 domains involved in the closure of the local sixfold opening and a thinner capsid shell during capsid maturation.

## 1. Introduction

Many viruses undergo drastic conformational changes and structural rearrangements before and after viral genome packaging [1,2,3]. Tailed bacteriophages, as the most diverse and extensive viruses in the Earth’s biosphere [2,4,5], are valued by researchers because they provide a simple model system which makes it easy to recognize the core biological processes related to all biology [6]. In many double-stranded DNA viruses, including herpesviruses, the formation of the tailed phages begins with the assembly of an empty precursor particle (procapsid) [2,7]. In general, the procapsid comprises an icosahedral round shell formed by a major capsid protein (coat protein) and a scaffolding protein or an equivalent scaffolding domain that is covalently attached to the coat protein [3,8,9,10]. One of twelve icosahedral vertices of the procapsid is replaced by a cylinder-shaped portal, which provides a channel for DNA packaging. During DNA packaging, the procapsid shell expands to a more angular intermediate to match the size of the viral genome. This process is accompanied by conformational changes in the coat protein and release of the scaffolding protein or domain [3,8,9,10].

The icosahedral capsid shell of the tailed phage, which is composed of hundreds of coat protein subunits, is relatively conserved. Facilitated by cryo-electron microscopy (cryo-EM) and X-ray crystallography, the capsid structures of many tailed phages have been determined at near-atomic resolutions [9,10,11,12,13,14]. Structural details of the tailed phages show the conserved folding of their coat proteins, which is defined as the HK97 fold because it was first discovered in phage HK97 [14,15]. These conserved domains include the A-domain (axial domain), P-domain (peripheral domain), E-loop (extended loop), and N-arm (N-terminal arm). There are some additional coat protein domains which appear in some, but not in all phages.

*Salmonella typhimurium* bacteriophage P22, a member of *Podoviridae*, is an established model system for studying protein assembly and conformational changes in macromolecular machines under different functional states [16,17]. Bacteriophage P22 is also a well-studied protein-cage model system, which is suitable for designing effective nanomaterial containers for use in programmed cargo encapsulation [18,19,20]. During the assembly of bacteriophage P22, the scaffolding protein acts as a catalyst of macromolecule assembly [21,22]. Approximately 250–300 copies of scaffolding protein gp8 and 415 copies of coat protein gp5 bind to each other to form the procapsid in the T = 7 *laevo* icosahedral arrangement [21,22]. In addition to the conserved HK97 fold, gp5 has a distinctive nonconserved domain (insertion domain or I-domain) that is inserted in the A-domain. In the procapsid and capsid, the gp5 subunits are arranged into pentons and hexons (collectively referred to as capsomers).

The structure of the P22 mature capsid has been resolved at 3.3 Å resolution [23]. In addition, the icosahedral procapsid structures of bacteriophage P22 have been extensively studied using cryo-EM from 3.8 Å to 28 Å resolutions [3,16,24,25,26]. Backbone models of the coat protein gp5 and the C-terminal region of the scaffolding protein gp8 built from a 3.8 Å cryo-EM map of the P22 procapsid [3] revealed that the C-terminal region of the scaffolding protein attaches to the inner surface of the procapsid around the icosahedral fivefold axis and the local sixfold axis. However, a later reported nuclear magnetic resonance (NMR) structure of the isolated I-domain of the P22 coat protein differed substantially from the gp5 backbone model [3,27]. Although the I-domain structure was used in conjunction with the 3.8 Å cryo-EM procapsid map to develop a refined model of the I-domain, as well as the full-length P22 coat protein, the amino acid residue registration of the model might be inaccurate due to the limited resolution of the cryo-EM map. Moreover, the high resolution structure involved in the interactions between scaffolding and coat proteins is still unknown, although the structure of the recombinant C-terminal region of the P22 scaffolding protein has been determined by NMR [22].

Here, we report the cryo-EM structures of the procapsid, empty procapsid, empty mature capsid, and mature capsid of phage P22 at resolutions of 2.6 Å, 3.9 Å, 2.8 Å, and 3.0 Å, respectively. The side chains of gp5 and the two C-terminal α-helices of gp8 were clearly resolved in the procapsid structure, allowing us to build accurate atomic models. Our structures of the procapsid and mature capsid at higher resolution reveal hydrogen bonds and salt bridges between the gp5 subunits in the procapsid and the structural changes involved in the closure of the local sixfold opening and a thinner capsid shell during capsid maturation.

## 2. Materials and Methods

### 2.1. Sample Purification

Salmonella typhimurium strain was grown in LB medium (10 g Tryptone, 5 g Yest extract, and 10 g sodium chloride per liter) for 5 h at 37 °C. The P22 phage (ATCC-97541-B1), which was purchased from the American Type Culture Collection (Manassas, VA, USA), was propagated on *S. typhimurium* for 4 h at 37 °C. After cell lysis, the culture continued for several hours to allow the growth of infected *S*. *typhimurium*. We separated and collected the bacteria and the supernatant using low-speed centrifugation at 6000× *g* for 15 min at 8 °C. The bacterial cells were lysed with 50% chloroform, and low speed centrifugation removed cell debris. Next, 1 M NaCl and 10% polyethylene glycol (PEG8000) (Amresco, Solon, OH, USA) were added to the supernatant, which was stored in an ice–water bath overnight. The precipitated P22 phage particles were resuspended in 10 mM Tris-HCl and 1 mM MgCl_2_ (pH 7.4) and then were purified by two rounds of continuous density centrifugation on CsCl cushions (CsCl) (Sigma, St. Louis, MO, USA). After the first centrifugation on 1.6 g/mL and 1.4 g/mL CsCl cushions at 100,000× *g* for 2 h at 8 °C, two bands were clearly visible (Figure S1A). The two bands were separated and collected by the second centrifugation on 1.6 g/mL and 1.4 g/mL CsCl cushions and dialyzed against 10 mM Tris-HCl and 1 mM MgCl2 (pH 7.4) overnight at 4 °C. The two bands of P22 particles were evaluated after negative staining with an electron microscope.

### 2.2. Cryo-EM Imaging

An aliquot of 3 μL from each of the two bands of P22 particles was applied to an amorphous nickel titanium alloy grid with carbon film, which was glow-discharged for 30 s. The grid was loaded into an FEI Vitrobot and the parameters were set as follows: a temperature of 8 °C, a relative humidity of 100%, and a blot time of 3.5 s. After completion of the sample blotting, the grid was plunged into solid–liquid ethane and transferred to liquid nitrogen. Cryo-EM images were collected by the Titan Krios G3i microscope equipped with a Gatan imaging filter and a K3 summit direct electron detector. The FEI EPU software proceeded to automatically collect data of the lower and upper bands at a magnification of 53,000× and 64,000×, corresponding to pixel sizes of 1.36 Å and 1.06 Å, respectively. The full dose of each movie of lower band and upper band of P22 particles was approximately 35 e^−^/Å^2^. Finally, 4668 movies was collected in total for the upper band, and 1000 movies for the lower band.

### 2.3. Image Processing of the P22 Particles

The icosahedral reconstructions of the P22 particles in the upper band (procapsid, empty procapsid, and empty capsid) were performed using our software [28,29] based on the common-line algorithms [30]. 

The defocus and astigmatism values of each image were determined by our software. The viral particles were boxed automatically using the program ETHAN [31], and these particles were manually classified into three types, namely, procapsid (small and full), empty procapsid (small and empty), and empty capsid (large and empty) (Figure S1B) for image reconstruction. The orientations and centers of the three types of particle images were determined by our software [28,29] based on common-line algorithms [30]. The icosahedral structures of the three types of particles were reconstructed by our reconstruction program ISAF imposing icosahedral symmetry [32]. The resolutions of the reconstructions were estimated by the Fourier shell correlation criterion [33].

To further improve the resolution of the structures, we applied local refinement and reconstruction to the asymmetric units of the procapsid, empty procapsid, and empty capsid by using local reconstruction (C1 symmetry). The local refinement and reconstruction were performed iteratively to improve the resolution until the orientations and centers in all datasets were stabilized, and the structural resolution could not be further improved. The P22 particle in the lower band (capsid) was reconstructed using the same approach.

### 2.4. Atomic Model Building and Refinement

Using COOT software [34], we manually built the atomic models of gp5 and gp8 on the basis of the cryo-EM density map of the procapsid. Furthermore, we refined the models through real-space refinement, as implemented in Phenix [35]. The refinement and validation statistics are presented in Table S1. All figures were prepared using UCSF Chimera [36] and ChimeraX [37].

## 3. Results

### 3.1. Cryo-EM Reconstruction of P22 Particles in the Two Bands of the Gradient

The bacteriophage P22 was propagated in *S. typhimurium*, and the phage lysate from the bacterial culture was purified using density gradient centrifugation. Two visible bands containing P22 particles were separated. The upper and lower bands were located at 1.4 g/mL- and 1.6 g/mL-CsCl cushions, respectively (Figure S1A). Cryo-EM analysis of the upper band showed that there were three types of P22 particles in the images, namely, small and full particles, small and empty particles, and large and empty particles (Figure S1B). We obtained the icosahedral structures of the three types of particles at 3.7 Å, 4.6 Å, and 4.0 Å resolutions (Figure S1C,D), respectively, by using cryo-EM and icosahedral reconstruction. The icosahedral structures of the three types of particles were virtually identical to the previously reported procapsid structure at 3.8 Å resolution [3], empty procapsid at 8 Å resolution [26], and capsid of the mature P22 at 3.3 Å resolution [23], respectively. We, therefore, designated them as procapsid, empty procapsid, and empty capsid, respectively (Figure S1C). 

**Figure 1:**
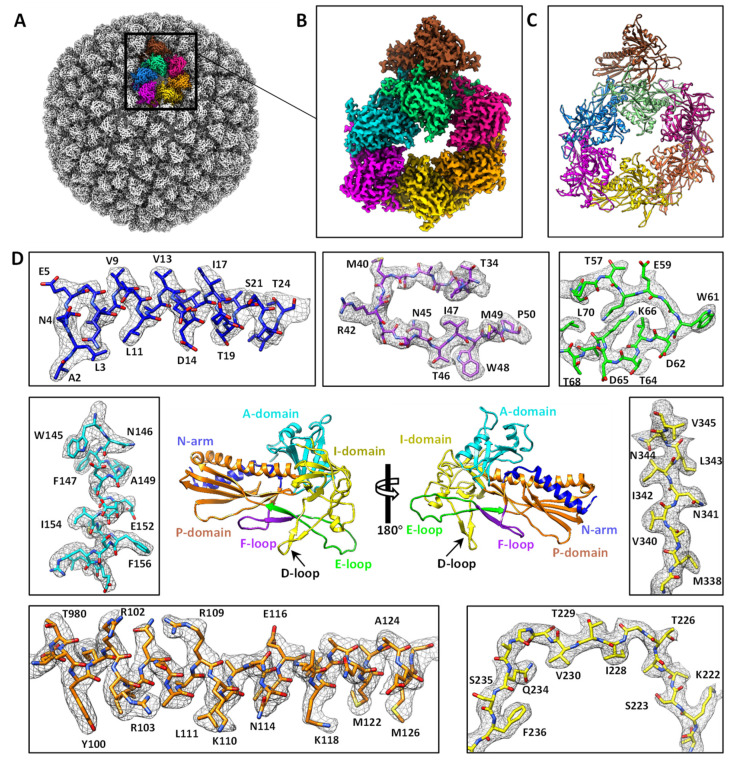
The cryo-EM structure of the P22 procapsid. (**A**) The overall structure of the procapsid. The seven subunits of the procapsid asymmetric unit are in different colors. (**B**) The segmented asymmetric unit from the local reconstruction of procapsid. (**C**) Accurate atomic models based on the density map in panel B. (**D**) Density maps of gp5 at 2.6 Å resolution superimposed on their atomic models. The gp5 structure is divided into six domains, namely, N-arm, P-domain, I-domain, A-domain, F-loop, and E-loop.

To further improve the resolution of the three structures, local reconstructions were performed of the asymmetric regions of the individual particles to improve the procapsid, empty procapsid, and empty capsid to resolutions of 2.6 Å, 3.9 Å, and 2.8 Å, respectively (Figures S1E, S2 and S3). These results revealed the imperfect icosahedral symmetry of the procapsid and the capsid. Based on the procapsid density map, we improved the atomic models for the seven subunits of the coat protein gp5 in the asymmetric unit of the procapsid protein (Figure 1 and Figure S2).

**Figure 2:**
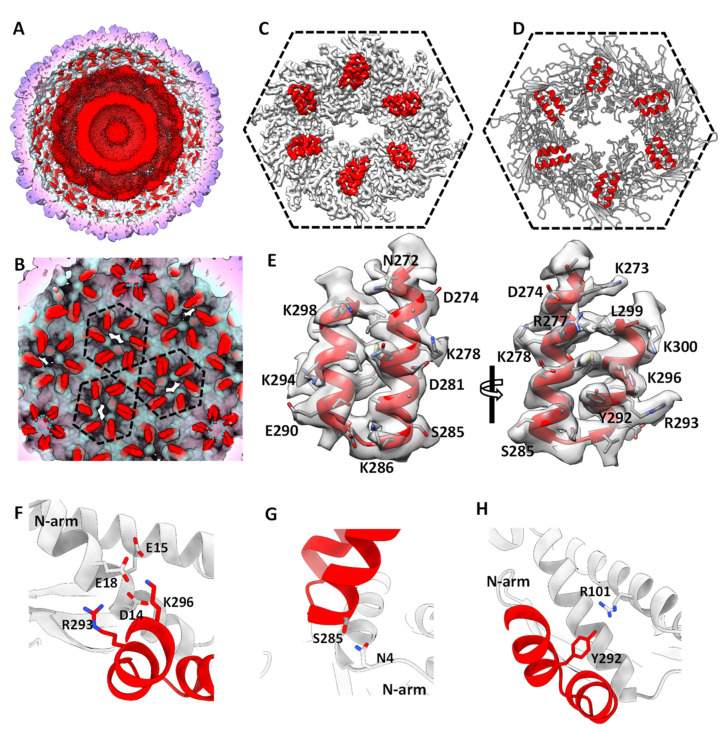
Interactions between the scaffolding protein and the inner procapsid. (**A**) The cut-open view of the procapsid structure. (**B**) The distribution of U-shaped helix-turn-helix motif (C-terminus of the scaffolding protein) on the inner surface of the procapsid. The two layers, which are attributed to the disordered scaffolding protein and ejection proteins, were removed computationally for clarity. (**C**) The hexon density segmented from the procapsid. The gp5 subunits and helix-turn-helix motif are in gray and red, respectively. (**D**) The atomic model of the density map in panel C. (**E**) An atomic model of the C-terminal residues of the scaffolding protein was built into the U-shaped helix-turn-helix density map. (**F**–**H**) Interactions between the helix-turn-helix motif and gp5.

The icosahedral structure of the empty procapsid was almost identical to that of the procapsid, except that the structure of the empty procapsid lacked the U-shaped structures within the capsid (Figure 2A,B and Figure S4), which was attributed to the C-termini of the scaffolding protein within the capsid [3,26] (described below). Except for the U-shaped structure, the densities within the procapsid were unstructured and revealed as two disordered concentric layers, which was attributed to the disordered scaffolding protein and ejection proteins in the procapsid (Figure 2A, Figures S1C and S4A).

Cryo-EM analysis of the lower band showed that there was only one type of particle (large and full) in the sample (Figure S1B). Icosahedral reconstruction of the large and full particle at 3.6 Å resolution (Figures S1C,D) showed that its structure was virtually identical to the previously reported mature P22 structure at 3.3 Å resolution [23]. 

### 3.2. Structure of the Coat Protein and Assembly of the Procapsid

There are seven subunits of the coat protein gp5 in the procapsid asymmetric unit, that is, six for the hexon and one for the penton. We built atomic models for the seven gp5 subunits based on our 2.6 Å resolution density map of the procapsid (Figure 1B–D and Figure S2). The superposition of the seven gp5 subunits showed root-mean-square deviation values ranging from 1.39 to 2.32 Å, suggesting that the seven subunits were almost identical (Figure S5). Compared with the previously reported Cα backbone model of the P22 procapsid gp5 [3] and the NMR structure of the gp5 I-domain [27], our structure provided a more accurate trace of the main chain and registration of the side chains (Figure 1D and Figure S2). The gp5 structure can be divided into six domains [3,27], namely, the N-arm (residues 2–30), P-domain (residues 31–33, 79–127, and 358–416), insertion (I-, residues 222–345), A-domain (residues 128–221, 346–357, and 417–430), E-loop (residues 51–78), and F-loop (residues 34–50) (Figure 1D). The N-arm domain is an N-terminal α-helix. The P-domain comprises a β-sheet flanked by a long α-helix. The A-domain comprises a β-sheet flanked by two short α-helices. Residues 194–208 of the A-domain are flexible (Figure S6). The I-domain in our procapsid gp5 structure differs from that in the previously reported Cα backbone model of the procapsid [3] in the trace of the main chain. By contrast, our I-domain structure is similar to the NMR structure of the isolated I-domain [27] in that they both have a β-barrel (Figure S7). However, the two loops varied markedly in the two structures: the D-loop (residues 239–254) became a β-hairpin in our structure, and the S-loop (residues 277–292) shifted (Figure S7). 

**Figure 3:**
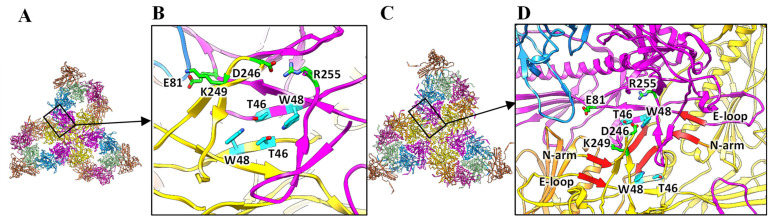
Inter-capsomere interactions among adjacent gp5 subunits in the procapsid and capsid. (**A**) A schematic representation of gp5 subunits around the threefold axis in the outer procapsid surface. (**B**) Salt bridges (E81-K249 and D246-R255) and a pair of hydrogen bonds (T46-W48) between neighboring gp5 subunits. (**C**) A schematic representation of gp5 subunits around the threefold axis in the outer capsid surface. (**D**) Interactions between coat subunits. The salt bridges and hydrogen bonds are disrupted upon capsid expansion, and β-augmentations (red) between neighboring coat proteins are formed.

A previous biochemical study showed that 18 temperature-sensitive mutations in gp5 could affect gp5 folding and procapsid assembly [38,39]. In addition to 10 of the 18 temperature-sensitive-folding (tsf) substitutions localized in the I-domain NMR structure [27], the remaining eight substitutions (W48Q, A108V, D174G, D174N, F353L, G403D, Y411H, and P418S) were also localized in our gp5 structure (Figure S8). In our structure, W48 was located in the F-loop, where it formed a hydrogen bond with T46 in the neighboring gp5 subunit (Figure 3A,B), which is consistent with the results of a previous biochemical study that reported the mutation W48Q to decrease the stability of the procapsid shell [38]. Among the remaining seven substitutions, A108, Y411, and G403 were located in the P-domain, whereas D174, F353, and P418 were located in the A-domain (Figure S8). These seven residues did not interact with the other gp5 subunits and, therefore, the decreased stability of these tsf mutants could intrinsically destabilize gp5, rather than disrupt interactions between gp5 subunits in the procapsid.

The gp5 subunits in the procapsid are closely linked to each other through electrostatic interactions. A previous structural study of P22 gp5 [16] based on a gp5 model generated from the NMR structure of the isolated gp5 I-domain [27] and the cryo-EM structure of the procapsid at 3.8 Å resolution [3] suggested that potential salt bridges between two neighboring gp5 subunits in the procapsid might occur between E81 and K249, as well as between R299 and D244 and R269 and D246. We identified the inter-subunit salt bridges between E81 and K249 in the two neighboring gp5 subunits, respectively, and between D246 and R255 in the two neighboring gp5 subunits, respectively (Figure 3B). The last two proposed salt bridges were not identified in our procapsid structure because their side chains projected toward different directions (Figure S9).

### 3.3. Interactions between the Scaffolding Protein and the Inner Procapsid

There were U-shaped structures of a helix-turn-helix motif anchored on the inner surface of the procapsid around the icosahedral fivefold axes and local sixfold axes (Figure 2A,B). The distribution of the U-shaped helix-turn-helix motif in our structure was similar to that of the previously reported Cα backbone model of the P22 procapsid [3], and each copy of the α-helix was associated with a gp5 subunit in the asymmetric unit in the same interaction mode. However, in our structure, the orientation of the U-shaped helix-turn-helix motif was reversed, with the “U” opening toward the fivefold or sixfold axes (Figure 2B–D). The quality of the U-shaped structure was further improved by the asymmetric averaging of the seven U-shaped structures in the asymmetric unit, allowing us to build an atomic model of the C-terminal residues (271–300) of the 303-residue scaffolding protein gp8 into the U-shaped structure (Figure 2C–E). The C-terminus of gp8 exhibited a helix-loop-helix motif, in which the main chain and the amino acid registration were almost identical to the coat-protein-binding domain of the previously reported NMR structure of gp8 (PDB ID: 2GP8) [22] (Figure S10A). The C-terminus of gp8 in the asymmetric unit was anchored to the inner capsid through electrostatic interactions mediated by R293 and K296 in the helix-loop-helix of gp8 and D14, E15, and E18 in the gp5 N-arm domain (Figure 2F and Figure S10B). In addition, S285 in gp8 formed a hydrogen-bond interaction with N4 in gp5 (Figure 2G and Figure S10C), and Y292 in gp8 formed a cationic π interaction with R101 in gp5 (Figure 2H and  Figure S10D); these interactions further stabilized the scaffolding protein in the capsid. 

Our structural results of the interactions between gp5 and gp8 in the procapsid are also evidenced by previous biochemical studies. The removal of 11 C-terminal residues of the P22 scaffolding protein abrogated its binding to the coat protein [40]. In addition, alanine-scanning mutagenesis in charged residues of the C-terminal region of gp8 and site-directed mutagenesis of gp8 and gp5 [41,42] suggest that R293 of gp8 bound to D14 of gp5, and K296 of gp8 formed the closest interaction with D14 and E18 of gp5. In a previous study, truncation mutants of gp8 showed that residues 280–294 of gp8 could interact with gp5 [43], which is consistent with the hydrogen bond interaction between S285 of gp8 and N4 of gp5 in our procapsid structure (Figure 2G). Moreover, E15 in gp5 maintains a weak interaction with gp8 as reported in a site-directed mutagenesis study [41], which is consistent with our structural results that the distance from E15 of gp5 to either R293 or K296 of gp8 is larger than the distance from either D14 or E18 of gp5 to either R293 or K296 of gp8 (Figure 2F and Figure S10B).

### 3.4. Conformational Changes in the Coat Protein during Viral Maturation

**Figure 4:**
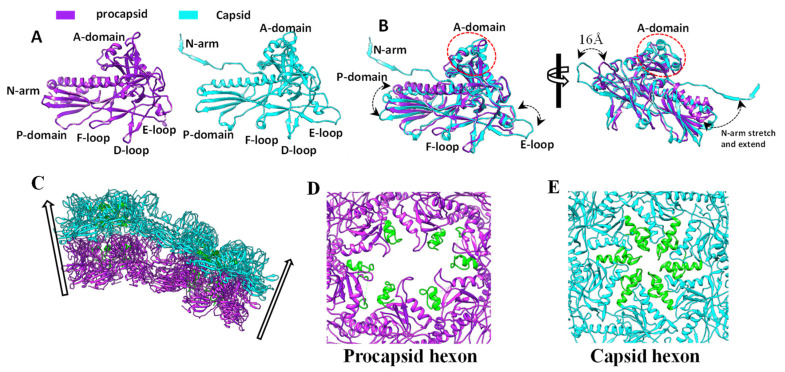
The conformational transition of the coat protein from the procapsid to the capsid. (**A**) Gp5 subunits of penton in the procapsid (left) and the capsid (right)**.** (**B**) The superposition of the coat protein subunit of the procapsid and capsid shows the conformational change during the expansion of the capsid. (**C**) A side view of the models of a hexon and a penton in the procapsid (purple) and the capsid (cyan) shows the capsid expansion. (**D**) The central opening of the hexamer of the procapsid. (**E**) The central opening of the hexamer of the capsid. The conformational change in the A-domain tip is colored in green.

Our structure of the mature P22 capsid was identical to the previously reported structure at 3.3 Å resolution [23]. Compared with the procapsid, the mature capsid expanded and became more angular, the sixfold opening closed, and the capsid became thinner. The structural changes of the capsid were caused by the conformational changes of gp5 (Figure 4, and Movie S1). The N-arm α-helix became a long loop that projected into the gp5 subunit in the adjacent asymmetric unit (Figure 4A,B and Figure S11A). The tip of the F-loop transformed into an α-helix (residues 36–42) (Figure 4A,B and Figure S11E). In the A-domain, one of the two short α-helices extended from residues 172–177 to 172–184 and the flexible loop (residues 190–208) became an α-helix and a fixed loop, resulting in the closure of the sixfold opening in the procapsid (Figure 4B,D,E, and Figure S11B). The P-domain and E-loop tilted, resulting in a thinner capsid shell (Figure S11C,D). The I-domain was largely unchanged, except for a minor shift of its D-loop (Figure S11E). The structural changes of these domains collectively gave rise to the expanded hexamer or pentamer (Figure 4C, Figure S1C and Movies S2–S4), resulting in an expanded mature capsid.

The salt bridges between E81 and K249, as well as those between D246 and R255 in the procapsid, and the hydrogen bond between W48 and T46 were disrupted due to the conformational change of gp5 (Figure 3C,D). Indeed, the effect of the tsf mutation (W48Q), which caused a decrease in the stability of the procapsid shells, was reduced upon capsid expansion [38]. New salt bridges were identified in the mature capsid as compensation. For example, E5, E15, and E159 formed salt bridges with K31, R42 and K216, respectively, in a neighboring gp5 subunit in the mature capsid [23]. In addition, the N-terminal arms of every subunit interacted with neighboring gp5 units through β-augmentation (Figure 3D). 

## 4. Discussion

The formation of the P22 procapsid requires 415 copies of the coat protein gp5 and approximately 250–300 copies of the scaffolding protein gp8, with a complex of portal and ejection proteins located at the unique fivefold vertex [44]. To improve the resolutions of the icosahedral structures of the P22 procapsid and capsid, we imposed icosahedral symmetry during the reconstruction. Therefore, the structure at the unique fivefold vertex was not resolved. Our structure showed that the two C-terminal α-helices of gp8 interacted with the N-arm of gp5, and the remaining regions of gp8 were unresolved, probably due to asymmetrical distribution of the remaining regions of gp8. Previous biochemical studies showed that gp8 plays important roles in the proper assembly of the procapsid [43,45]. 

Although our structure showed that each gp5 subunit was associated with a gp8 subunit, the copy number of the gp5 and gp8 in the procapsid [21] indicates that some gp5 subunits are not associated with the scaffolding protein. This partial occupancy of gp8 in the procapsid is evidenced in our procapsid structure, which showed that the density of the gp8 subunit was obviously weaker than that of the gp5 subunit. This observation suggests that the distribution of the gp8 subunits in the procapsid is an artifact resulting from the imposed icosahedral symmetry during the reconstruction. Our results revealed that the structure of the empty procapsid without gp8 was identical to that of the procapsid, suggesting that the release of gp8 alone is not sufficient to trigger the conformational changes in gp5.

Capsid expansion and maturation are achieved by conformational changes in gp5. The most dramatic change occurred in the coat N-arm domain, in which the N-terminal α-helix became the extending loop. A similar conformational change upon maturation also occurred in phages T4, T5, T7, SPP1, P23–45, and lambda [9,10,11,46,47] with different triangle numbers and capsid sizes (Figure S12), suggesting that this structural change is conserved in the tailed phages, and is independent of the triangle number and capsid size. In addition, the tilts of the P-domain and E-loop were also conserved in these phages.

The A-domain of gp5 is also involved in capsid expansion and maturation. The A-domain tip (residues 158–209) flips and closes the sixfold opening during the transition from the procapsid to the mature capsid. A similar conformational change in the A-domain also occurred in the coat proteins of phages SPP1, T5, and T7 [9,10,46] (Figure S13). However, the local sixfold axes in both procapsids and capsids of phages T4, lambda, and HK97 [12,13,47,48] were closed (Figure S14). Phages P22, SPP1, T7, T4, and lambda [3,9,10,49,50] encode scaffolding proteins for procapsid assembly. Among these phages, the scaffolding proteins of P22, SPP1, and T7 [3,9,10] are released through the opening of the hexamer during the maturation of the capsid. It is reported that the P22 scaffolding protein can participate in capsid assembly again, that is, after it is released [21]. Presumably, T7 and SPP1 also recycle their scaffolding proteins. By contrast, phages T4 [51] and lambda [50] remove scaffolding proteins by the viral encoded proteases after capsid maturation. The mechanism of protease digestion of these phages explains why they do not have a central opening in the hexamer. Notably, phages T5 [46,52] and HK97 [53] do not encode the scaffolding protein, but rather use the N-terminal domain of the major capsid protein as the scaffolding protein, which is also digested by the viral-encoded protease during the maturation of the head. The reason why the local sixfold axis of the HK97 procapsid is closed while that of T5 procapsid is open remains to be elucidated, although both phages use the mechanism of protease digestion to remove the scaffolding protein during the capsid maturation.

## Data Availability

The electron density maps and atomic coordinates have been deposited in the EM Data Bank (accession no. EMD-35120, EMD-35121, EMD-35123, EMD-35124, EMD-35126, EMD-35127, EMD-35132, and EMD-35133), and Protein Data Bank (ID codes 8I1T and 8I1V).

## Supplementary Materials

The following supporting information can be downloaded at: https://www.mdpi.com/article/10.3390/v15020355/s1, Figure S1: Cryo-EM images, density maps, and Fourier shell correlation (FSC) curves; Figure S2: Density maps (transparent or mesh) of the coat protein gp5 in the procapsid are superimposed in the atomic models; Figure S3: Density maps of the coat protein gp5 of th eprocapsid, empty procapsid, and empty capsid by using icosahedral (left) and local reconstructions (right); Figure S4: The structural comparison between the procapsid and the empty procapsid; Figure S5: All seven subunits of the coat protein in the asymmetric unit of the procapsid; Figure S6: Density map of gp5 superposed on its atomic model; Figure S7: Structural comparison of the I domain in our procapsid with the previously reported isolated I domain; Figure S8: Temperature-sensitive mutations are mapped in gp5 in our procapsid; Figure S9: Side chains of two putative salt bridges (D246-R269 and D244-R299) in two neighboring gp5 subunits (magenta and yellow), which correspond to subunits C and D in Figure S5; Figure S10: Structure of the C-terminus (residues 271–300) of the scaffolding protein gp8; Figure S11: Conformational changes of the coat protein gp5 from the procapsid(purple) to the capsid (cyan); Figure S12: Conformational changes upon maturation in phages T4, T5, T7, SPP1, P23-45, and lambda; Figure S13: In phages SPP1, T5, and T7, the A-domain tip flips and closes the sixfoldopening during the transition from the procapsid to mature capsid; Figure S14: For phages T4, lambda, and HK97, the local sixfoldaxes in both the procapsidand the capsid are closed; Table S1: Refinement and model statistics; Movie S1: The structural changes of the capsid were caused by the conformational changes of gp5; Movie S2–S4: The structural changes of these domains collectively gave rise to the expanded hexamer or pentamer.
