# Supplementary material for: Assembly and Capsid Expansion Mechanism of Bacteriophage P22 Revealed by High-Resolution Cryo-EM Structures
Source: Viruses. 2023 Jan 26;15(2):355. doi: 10.3390/v15020355 (PMC9965877; doi:10.3390/v15020355)
Supplement: Supplementary file 1 [file viruses-15-00355-s001.zip › SI Figures.pdf]

# Supplementary Figure

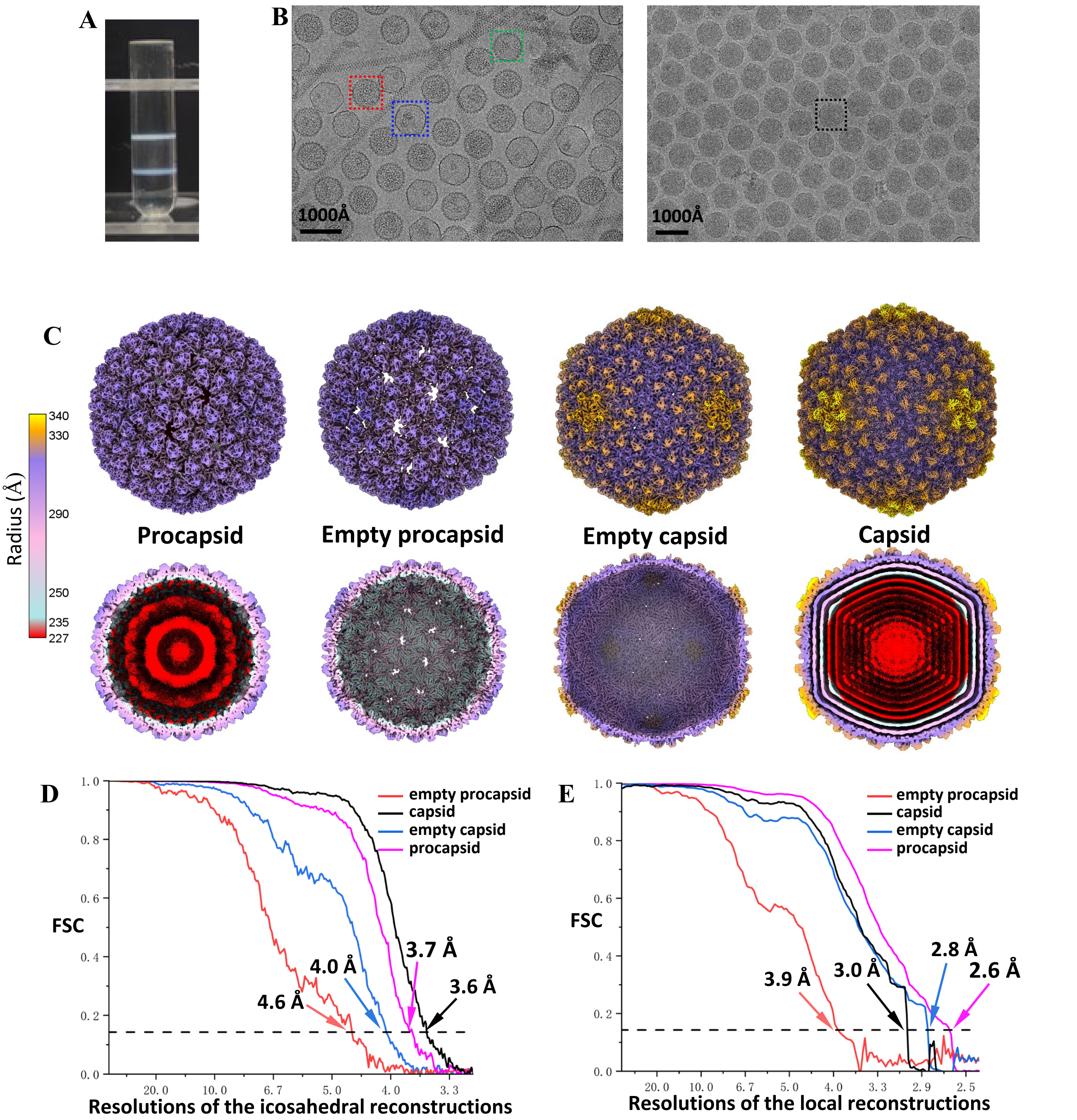


**Figure S1. Cryo-EM images, density maps, and Fourier shell correlation (FSC) curves. (A)** Two bands containing P22 particles were separated by density gradient centrifugation. **(B)** Cryo-EM images of P22 upper- (left) and lower-bands (right). A small and full particle, a small and empty particle, and a large and empty particle are enclosed in red, green, and blue boxes, respectively (left). A large and full particle is enclosed in a black box (right). **(C)** Density maps of the P22 procapsid, empty procapsid, empty capsid and capsid. **(D and E)** Structural resolutions of the icosahedral and local reconstructions of the procapsid, empty procapsid, empty capsid, and capsid.

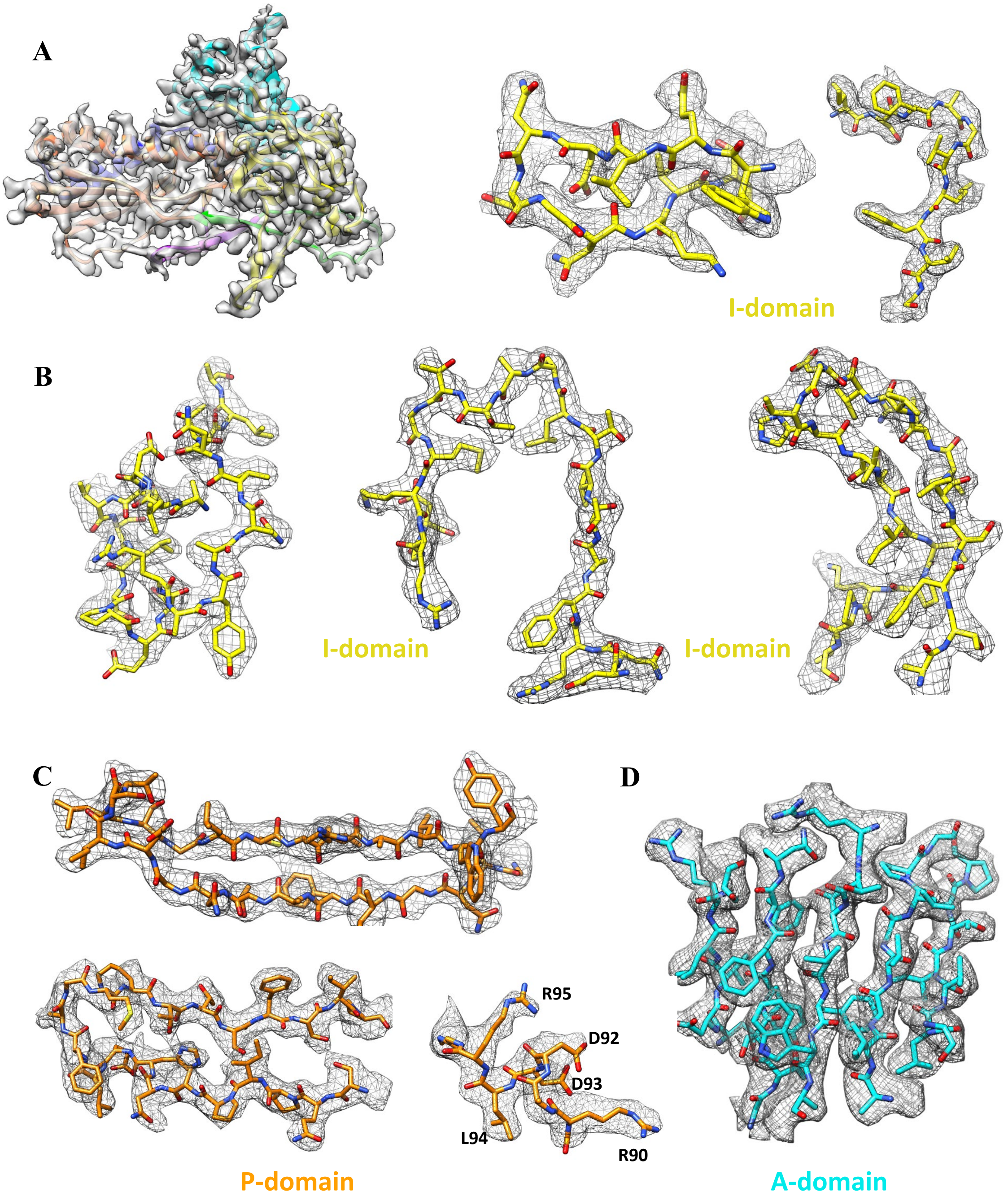


**Figure S2. Density maps (transparent or mesh) of the coat protein gp5 in the procapsid are superimposed in the atomic models.**

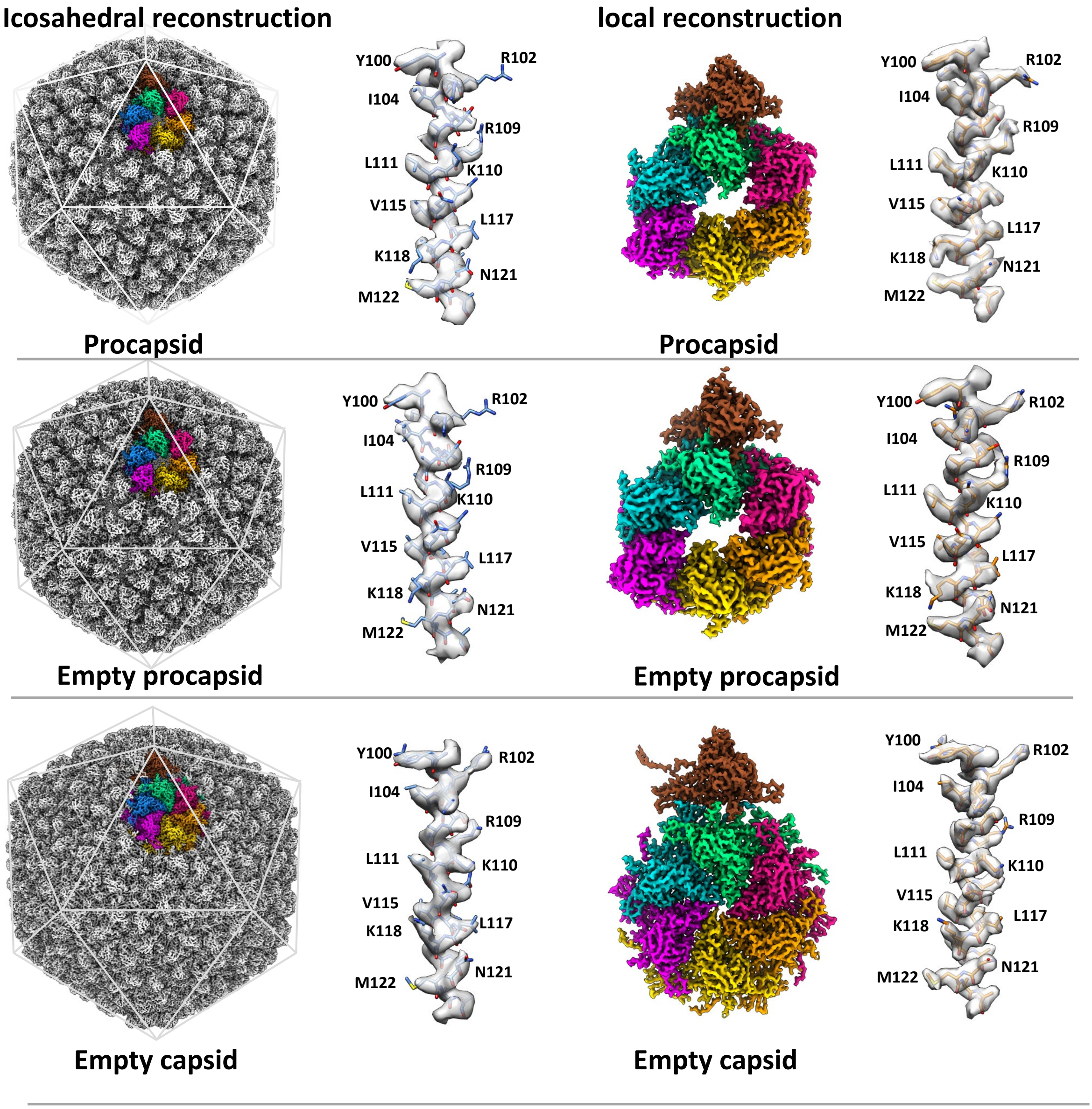


**Figure S3. Density maps of the coat protein gp5 of the procapsid, empty procapsid, and empty capsid by using icosahedral (left) and local reconstructions (right).**

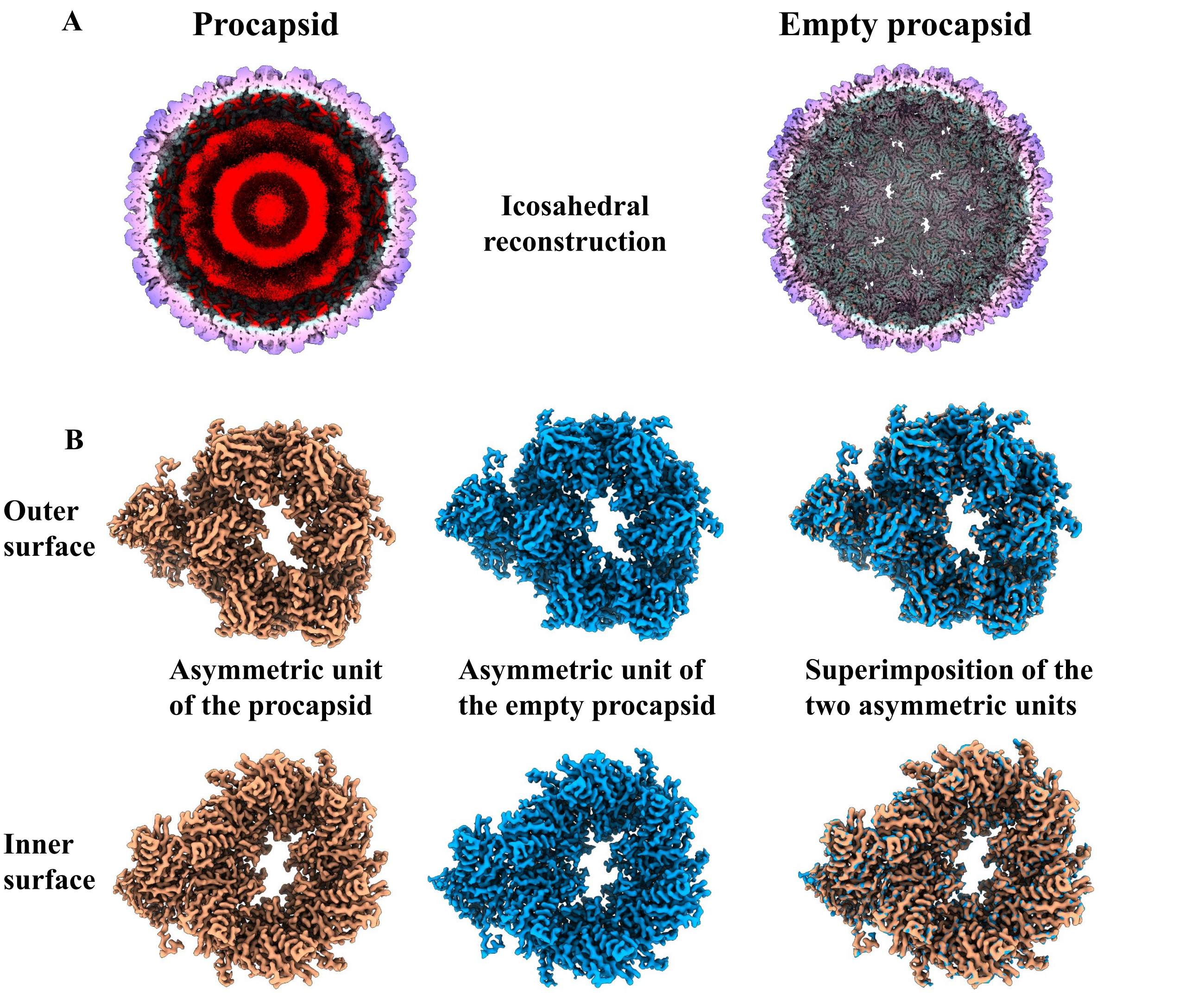


**Figure S4. The structural comparison between the procapsid and the empty procapsid. (A)** Cut-open views of the procapsid (left) and the empty procapsid (right). **(B)** Asymmetric unit density maps of the procapsid (brown) and the empty procapsid (blue). Outer and inner surfaces of the asymmetric units are shown (top and bottom rows respectively). The superimposition of the two asymmetric units shows that the two structures are identical (right column).

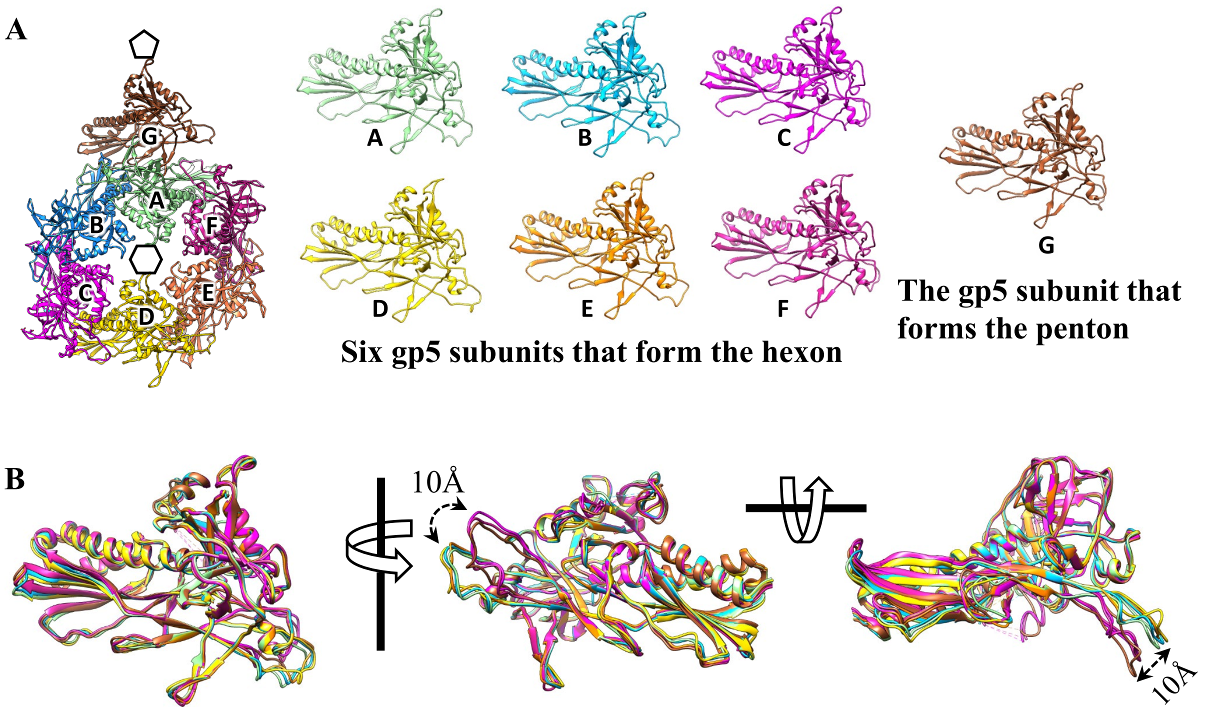


**Figure S5. All seven subunits of the coat protein in the asymmetric unit of the procapsid. (A)** Six gp5 subunits A-F that form the hexmer and the gp5 subunit G that forms the pentamer in the procapsid. **(B)** All seven subunits in the asymmetric unit of the procapsid are superimposed. The overlapping this models reveals the structural variations in the seven gp5 subunits. There are two conformations for the E-loop, approximately 10 angstroms apart as labeled (right). To adapt curvature of the procapsid, the E-loop of the gp5 subunits A, B D, and E adopt one conformation, and C, F, and G adopt another conformation.

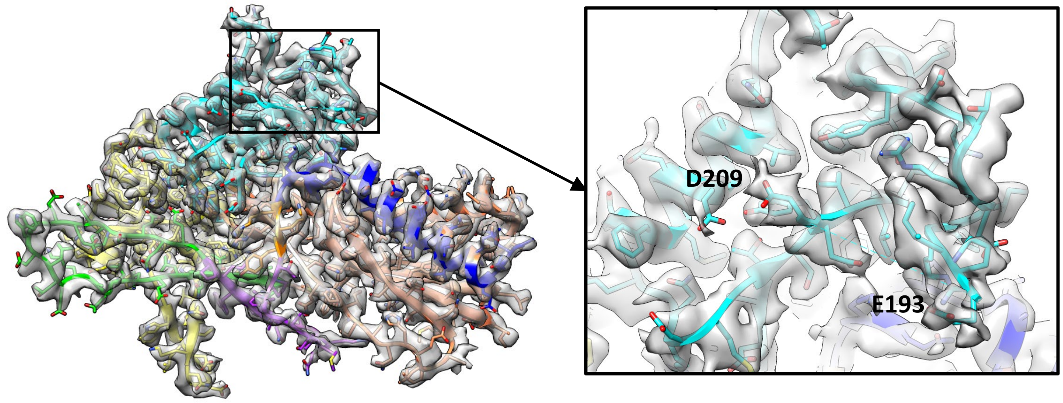


**Figure S6. Density map of gp5 superposed on its atomic model. The residues 194–208 of the A-domain were not resolved.**

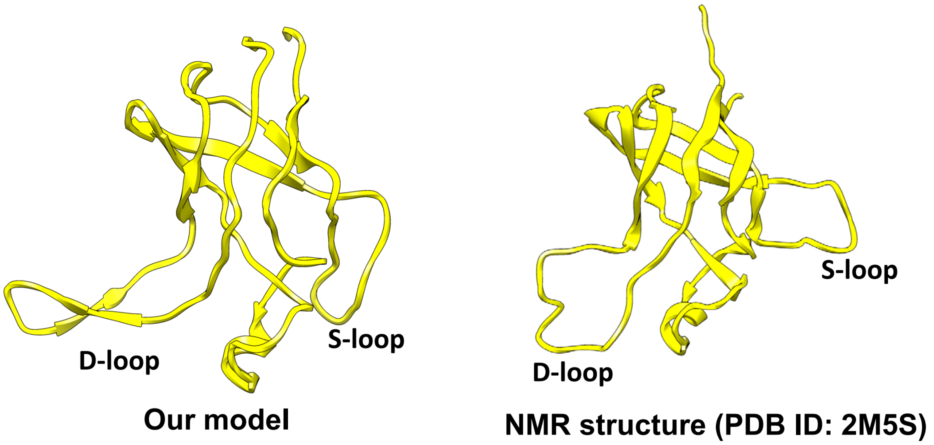


**Figure S7. Structural comparison of the I domain in our procapsid with the previously reported isolated I domain.** Structures of our gp5 I domain (left) and the isolated I domain (right, PDB ID: 2M5S).

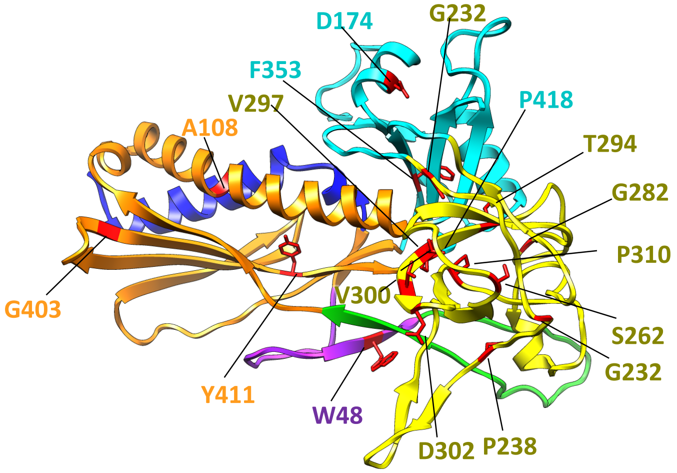


**Figure S8. Temperature-sensitive mutations are mapped in gp5 in our procapsid. The color scheme of the domains is identical to that in Fig. 1D.**

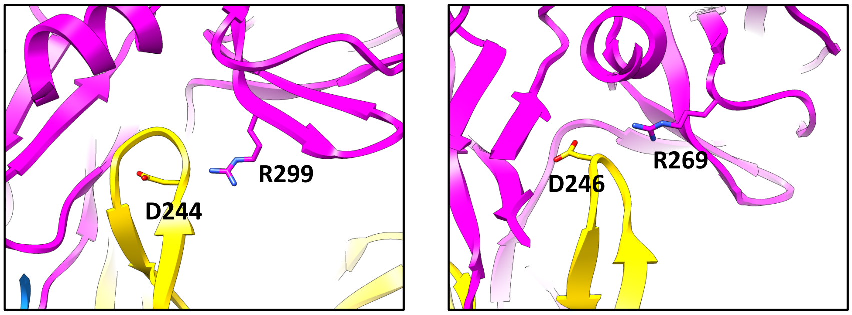


**Figure S9. Side chains of two putative salt bridges (D246-R269 and D244-R299) in two neighboring gp5 subunits (magenta and yellow), which correspond to subunits C and D in Figures S5.** They were proposed based on the gp5 model generated from the NMR structure of the isolated gp5 I-domain and the cryo-EM structure of the procapsid, project towards different directions in our structure.

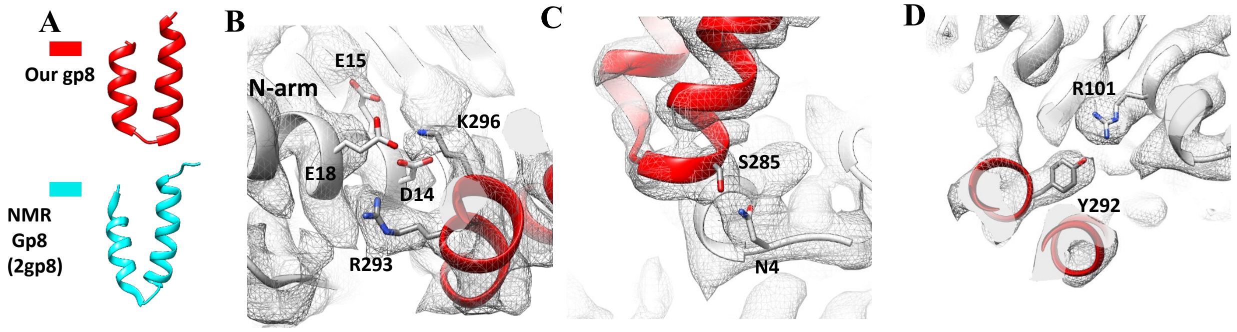


**Figure S10. Structure of the C-terminus (residues 271-300) of the scaffolding protein gp8. (A)** The NMR structure (cyan, PDB ID: 2GP8) and our structure (red)**. (B-D)** Interactions between gp5 and the C-terminus of gp8. Density maps (transparent) are superimposed on their models, respectively. The gp5 and gp8 models are in gray and red, respectively.

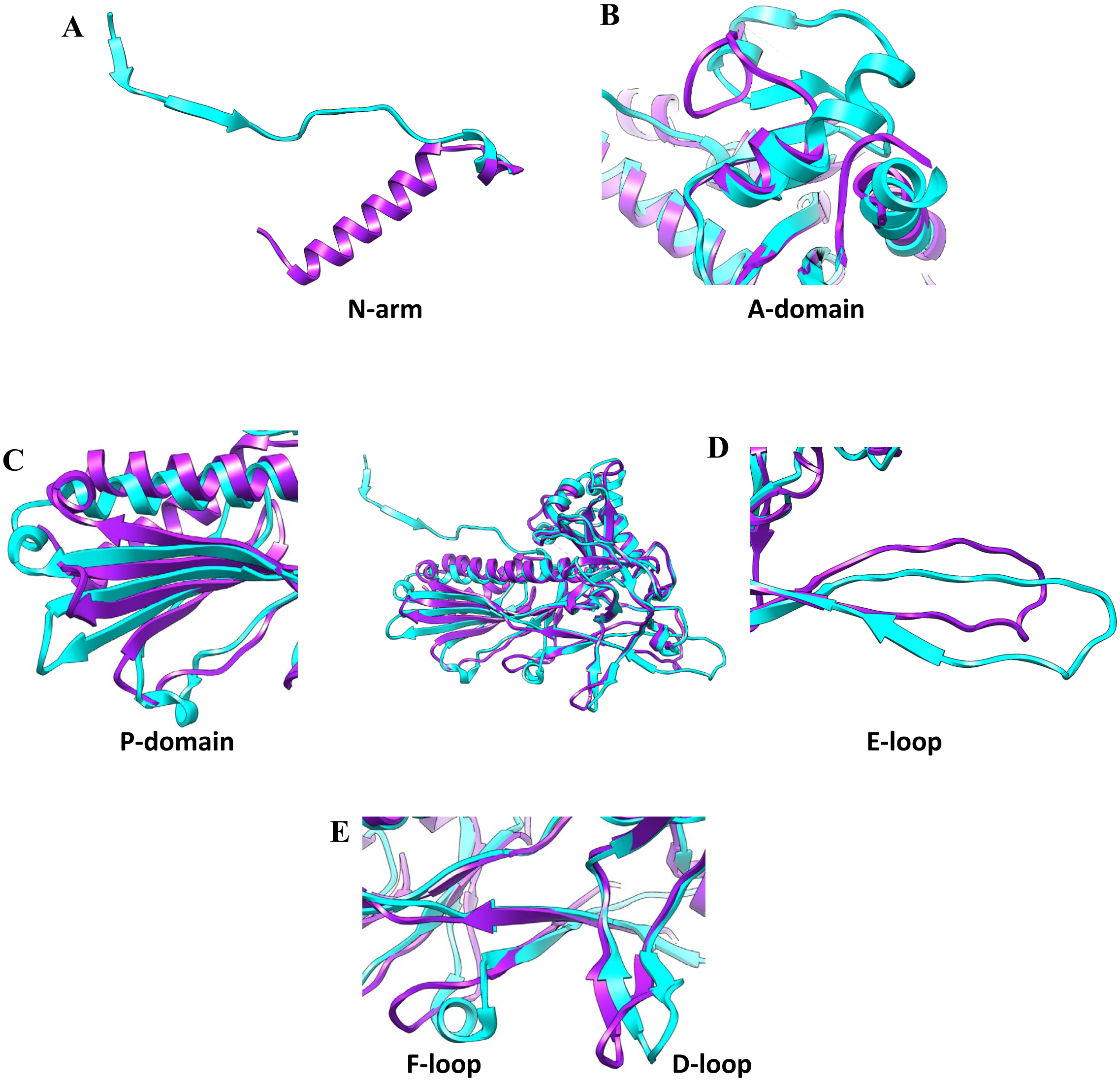


**Figure S11**. **Conformational changes of the coat protein gp5 from the procapsid (purple) to the capsid (cyan).**

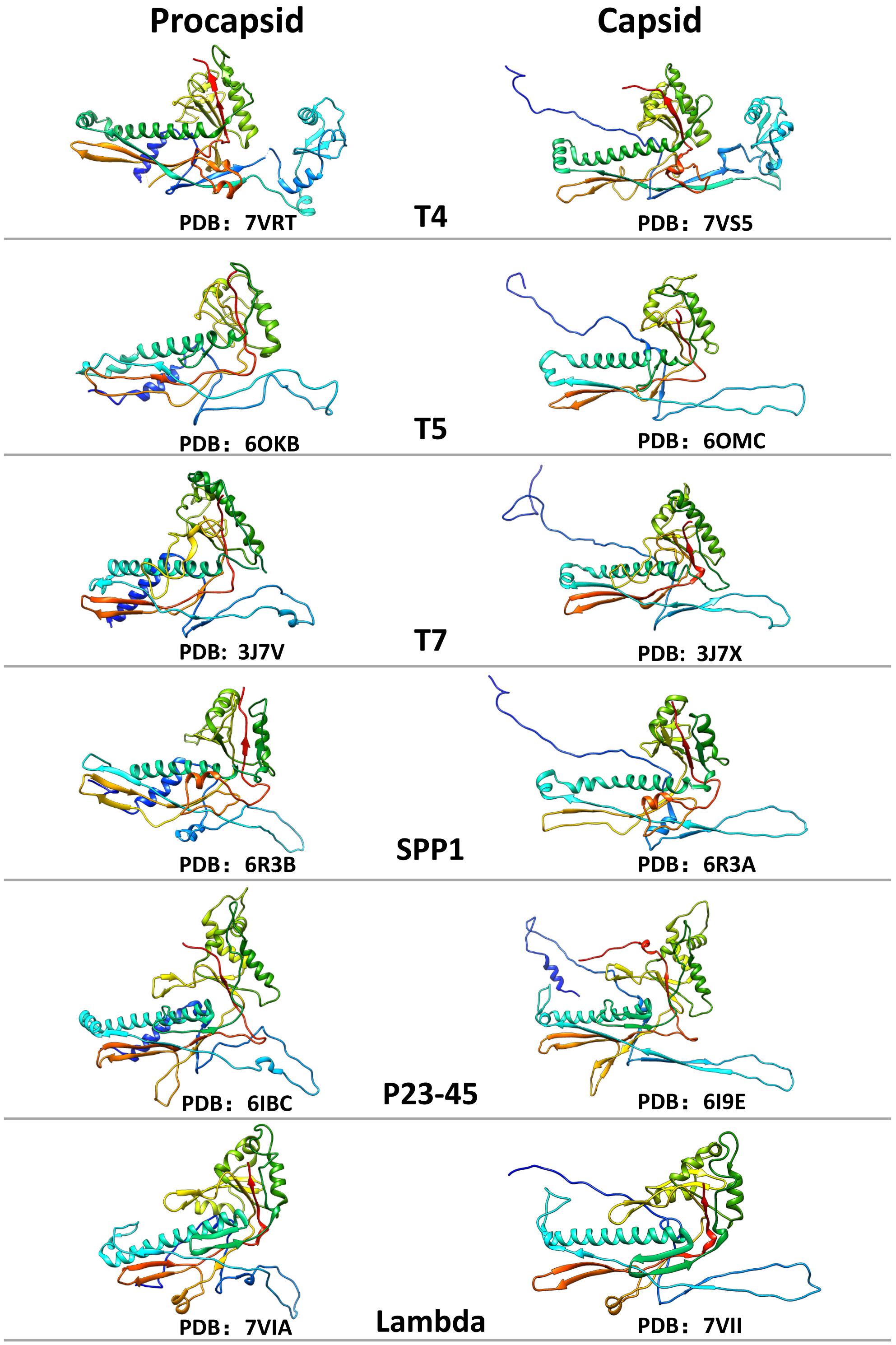


**Figure S12. Conformational changes upon maturation in phages T4, T5, T7, SPP1, P23-45, and lambda.**

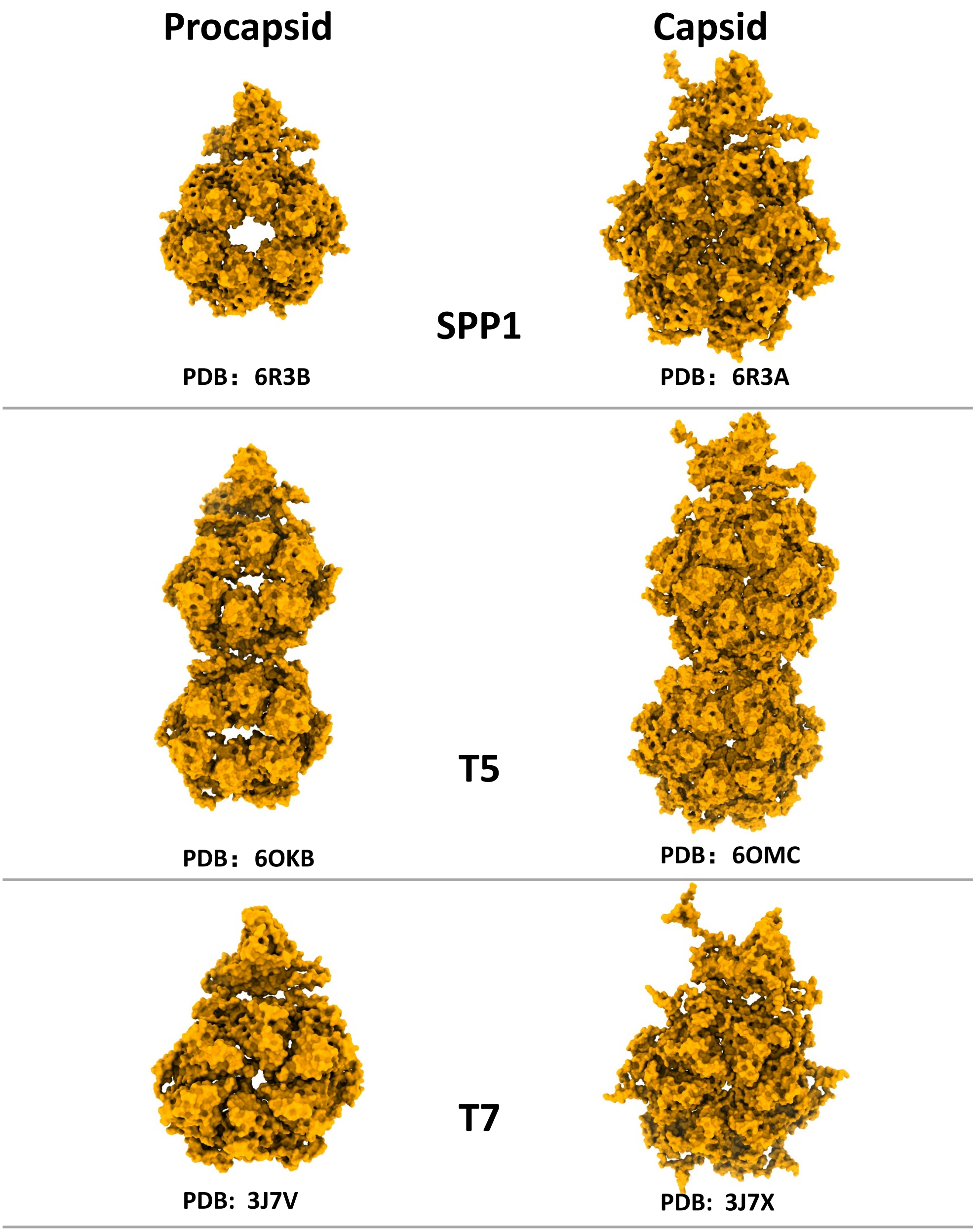


**Figure S13. In phages SPP1, T5, and T7, the A-domain tip flips and closes the sixfold opening during the transition from the procapsid to mature capsid. These atomic models were displayed using surface rendering in UCSF ChimeraX.**

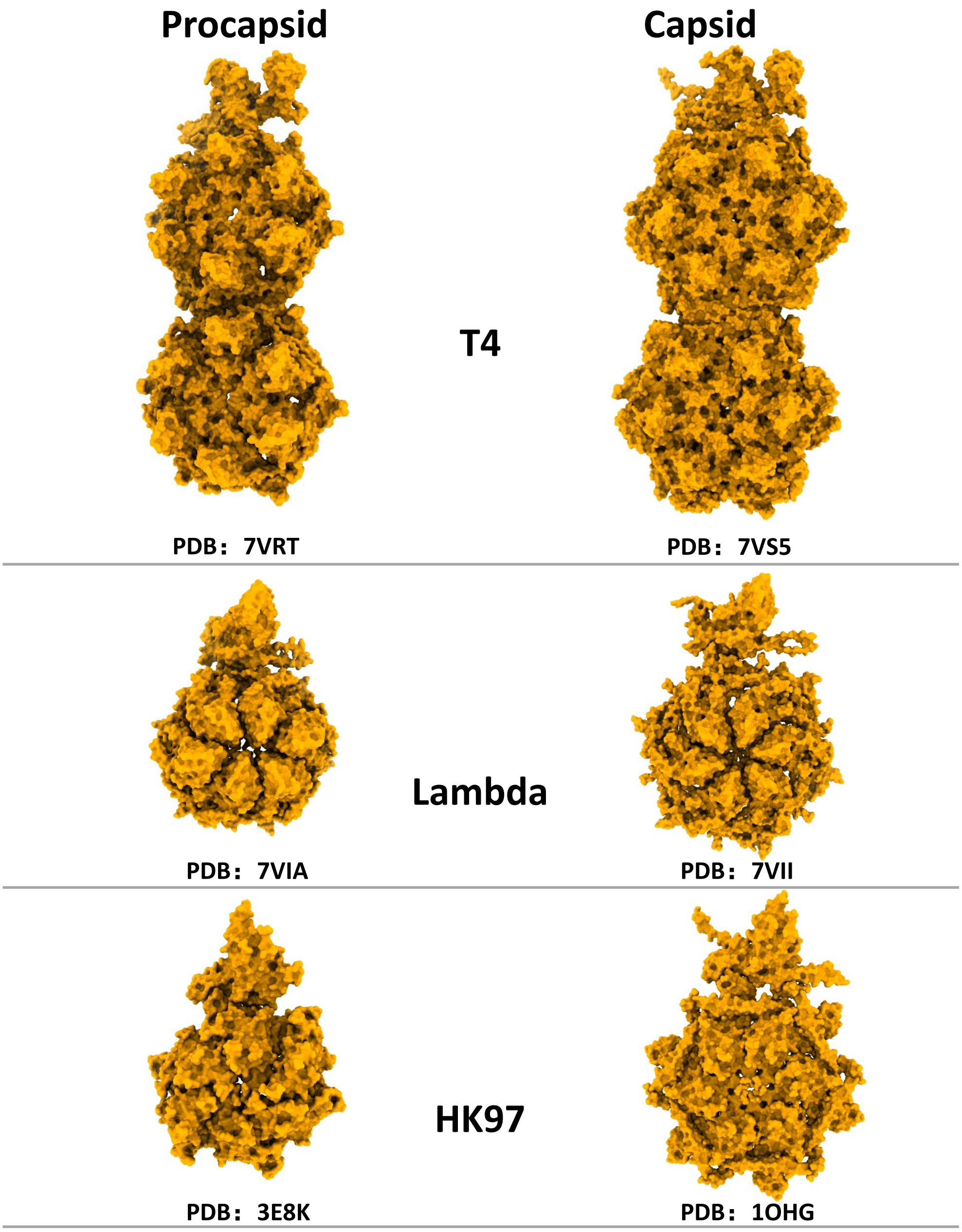


**Figure S14. For phages T4, lambda, and HK97, the local sixfold axes in both the procapsid and the capsid are closed. These atomic models were displayed using surface rendering in UCSF ChimeraX.**

## Table S1 Refinement and model statistics.

| Data collection | | | | |
|---|---|---|---|---|
| Electron microscopy | FEI 300 kV Titan Krios G3i, K3 camera | | | |
| Pixel size((Å)) | 1.06 | | 1.36 | |
| Defocus range | 1.6 to 2.4um | | | |
| Total movie-mode micrographs | P22 procapsid,empty procapsid, empty capsid | | P22 mature capsid | |
| | 4,668 | | 1,000 | |
| **Icoshadral reconstruction（I2 symmetry)** | | | | |
| | procapsid | empty procapsid | empty capsid | capsid |
| Total particles | 22,169 | 1257 | 7002 | 37914 |
| Resolution(Å) | 3.7 | 4.6 | 4 | 3.6 |
| EMDB ID | EMD-35126 | EMD-35124 | EMD-35120 | EMD-35132 |
| **Local reconstruction（C1 symmetry)** | | | | |
| | procapsid | empty procapsid | empty capsid | capsid |
| | asymmetry unit | asymmetry unit | asymmetry unit | fivefold-axes |
| Resolution(Å) | 2.6 | 3.9 | 2.8 | 3 |
| B-factors | 90 | 90 | 100 | 120 |
| EMDB ID | EMD-35123 | EMD-35127 | EMD-35121 | EMD-35133 |
| **Atomic models refinement/statistics (phenix.real_space_refine）** | | | | |
| | P22 procapsid | | P22 empty capsid | |
| Protein | gp5 and gp8 | | gp5 | |
| PDB ID | 8I1V | | 8I1T | |
| Model Resolution in Refinement(Å) | 2.6 | | 2.8 | |
| Total Residues | 2920 | | 2904 | |
| CC (model to map fit) | 0.8133 | | 0.8486 | |
| Ramachandran most favorable (%) | 97.23 | | 96.45 | |
| Ramachandran additionally allowed (%) | 2.77 | | 3.31 | |
| Ramachandran disallowed (%) | 0 | | 0.23 | |
